# Supplementary material for: A Graded Approach for Evaluating Health Claims about Plant-Based Food Supplements: Application of a Case Study Methodology
Source: Nutrients. 2021 Aug 2;13(8):2684. doi: 10.3390/nu13082684 (PMC8399096; doi:10.3390/nu13082684)
Supplement: Supplementary file 1 [file nutrients-13-02684-s001.zip › nutrients-1317274-supplementary.pdf]

Supplementary Material

Studies classification
Human studies are globally divided in three categories: experimental studies, quasi-experimental studies and non-experimental studies called also observational studies.

Experimental studies (RCT)
The experimental studies correspond to the clinical trials among which the gold standard is the controlled, randomised, double blind vs. placebo or versus reference treatment trial. Controlled means that the experimental conditions are fixed and in particular patients's selection criteria and their follow up conditions. Randomised means that the treatment given to each subject is not decided by the practitioner but predetermined in advance by a randomisation list in order to avoid that the choice of the practitioner may interfere with the evaluation of the studied product efficacy. Double blind means that the two products have exactly the same appearance and that either the practitioner or the patient does not know if they are receiving the new treatment, the placebo or the reference treatment. This trial is generally conducted on two parallels arm that means two groups of patients receiving the studied treatment or the placebo or the reference treatment. It can be also conducted according a cross over design. In a cross over design, the study is carried on two successive phases separated by a wash out period. During the first phase the patient take the studied product or the comparator and then the comparator if they have taken the studied product at the first phase or the contrary. The advantage of such a plan is that subjects are compared to themselves and not to another group of subjects and because the intra-individual variance is lower than the inter-individual variance it strongly reduces the sample size required. On the other hand, it requires that at the end of the washout period, the studied parameter of the subject is again strictly comparable to its baseline at the inclusion visit which is a strong limitation to its use.

Quasi-experimental studies
The main difference between the previous one is linked to the fact that they are non-randomised studies. These designs are frequently used when randomisation is not feasible due to logistically or ethical reasons. Quasi-experimental design encompasses a broad range of intervention studies aiming to demonstrate causality between an intervention and an outcome. Changes of a biomarker before and after the intervention is one of an example of quasi-experimental study without control group. When before/after measures (or pre-test/post-test design) are repeated and spaced at equal interval of time, this design corresponds to interrupted time-series designs. Quasi-experimental designs could also involve control group with or without pre-tests. Nevertheless, without randomisation, there is a high probability that treatment and the comparator groups are not comparable, that minors the internal validity of the trial. Statistical analysis will have to take into account this point through adjusted analysis on the criteria, which statistically differs from each other. The comparator group could be in order to reduce the potential biases, rather to let the practitioner to make his choice of the treatment, in many quasi experimental trial, each practitioner or each centre is using only one treatment which will be given to all the patient included in the study. This corresponds to what is called cluster trials.

Non-experimental or observational studies
These studies tend to reflect the real life i.e. the real conditions in which the product is prescribed by the practitioner and used by the subjects. The retrospective one or the prospective one is also called cohort studies and allows an intrinsic description of the evolution of patients under the studied treatment without comparisons with other treatments. The main difficulties of the retrospective cohort are linked to the difficulties to get the data in subject records which have not been specifically organised for the studies and the bias memory when patients are questioned retrospectively. The prospective cohort is more informative and are more and more often required by health authorities to complete experimental

studies to evaluate if the benefit demonstrated in experimental conditions is maintained in real life conditions.
